# Biventricular adaptation to volume overload in mice with aortic regurgitation

**DOI:** 10.1186/1532-429X-11-27

**Published:** 2009-08-11

**Authors:** Christopher J Berry, Jordan D Miller, KellyAnn McGroary, Daniel R Thedens, Stephen G Young, Donald D Heistad, Robert M Weiss

**Affiliations:** 1 Division of Cardiovascular Medicine, University of Iowa Carver College of Medicine, Iowa City, IA, USA; 2 Division of Cardiovascular Surgery, Mayo Clinic and Foundation, Rochester, MN, USA; 3 Department of Radiology, University of Iowa Carver College of Medicine, Iowa City, IA, USA; 4 Departments of Medicine and Human Genetics, David Geffen School of Medicine, University of California Los Angeles, USA

## Abstract

**Background:**

Aortic valve regurgitation is usually caused by impaired coaptation of the aortic valve cusps during diastole. Hypercholesterolemia produces aortic valve lipid deposition, fibrosis, and calcification in both mice and humans, which could impair coaptation of cusps. However, a link between hypercholesterolemia and aortic regurgitation has not been established in either species. The purpose of this study was to ascertain the prevalence of aortic regurgitation in hypercholesterolemic mice and to determine its impact on the left and right ventricles.

**Methods and Results:**

Eighty *Ldlr*^-/-^/*Apob*^100/100^/*Mttp*^fl/fl^/Mx1Cre^+/+ ^("Reversa") hypercholesterolemic mice and 40 control mice were screened for aortic regurgitation (AR) with magnetic resonance imaging at age 7.5 months. The prevalence of AR was 40% in Reversa mice, with moderate or severe regurgitation (AR^+^) in 19% of mice. In control mice, AR prevalence was 13% (p = 0.004 *vs*. Reversa), and was invariably trace or mild in severity. In-depth evaluation of cardiac response to volume overload was performed in 12 AR-positive and 12 AR-negative Reversa mice. Regurgitant fraction was 0.34 ± 0.04 in AR-positive *vs*. 0.02 ± 0.01 in AR-negative (mean ± SE; p < 0.001). AR-positive mice had significantly increased left ventricular end-diastolic volume and mass and reduced ejection fraction in both ventricles. When left ventricular ejection fraction fell below 0.60 in AR-positive (*n *= 7) mice, remodeling occurred and right ventricular systolic function progressively worsened.

**Conclusion:**

Hypercholesterolemia causes aortic valve regurgitation with moderate prevalence in mice. When present, aortic valve regurgitation causes volume overload and pathological remodeling of both ventricles.

## Background

Mild chronic aortic regurgitation occurs with a prevalence of about 9% in women and 13% in men[1]. Moderate or severe aortic regurgitation is less common, but the prevalence doubles with each decade of advancing age. The clinical course of patients with aortic regurgitation is determined by the severity of valve regurgitation as well as the extent to which ventricular remodeling compensates for volume overload[2,3].

Aortic regurgitation is usually caused by defective coaptation of valve cusps during diastole, which can arise from a variety of diseases of the valve itself or the aortic root[4]. In humans, hypercholesterolemia is a risk factor for aortic stenosis and aortic sclerosis, but a causal link with aortic regurgitation has not been established[1]. Hypercholesterolemic Reversa mice (*Ldlr*^-/-^/*Apob*^100/100^/*Mttp*^fl/fl^/Mx1Cre^+/+^) [5] develop oxidant stress in the aortic valve [6] and a programmed injury response leading to valve calcification[7,8].

In the current study, we tested the hypotheses that hypercholesterolemia causes aortic valve regurgitation in mice, and that ensuing volume overload leads to pathological remodeling of both ventricles.

## Methods

### Mice

Reversa mice (*Ldlr*^-/-^/*Apob*^100/100^/*Mttp*^fl/fl^/Mx1Cre^+/+^) have been described previously[5]. In brief, these are severely hypercholesterolemic mice in which the hypercholesterolemia can be "switched off" by Cre-mediated inactivation of the gene for microsomal triglyceride transfer protein. Beginning 1.5 months of age, Reversa mice received a Western diet (#7088137, Harlan-Tiklad, 42% of calories from fat, 0.25% cholesterol), leading to average total cholesterol ~900 mg/dl. At age 7.5 months, 80 Reversa mice (45 males and 35 females) and 40 normocholesterolemic control mice were screened by cardiovascular magnetic resonance (CMR) for aortic regurgitation. The control group included C57BL/6 mice (*n *= 19); superoxide dismutase knockout mice (*n *= 10); interleukin-10 knockout mice (*n *= 7), and Reversa mice in which the hypercholesterolemia had been eliminated by inducing the *Cre *transgene with an injection of polyinosinic-polycytidylic ribonucleic acid (pIpC) at age 1 month (*n *= 4). Control mice were fed normal chow throughout the study period. We studied a genetically diverse group of control mice to exclude the possibility that findings in Reversa mice were due to a "passenger gene" rather than the hypercholesterolemia *per se*. All experimental procedures were approved by the Office of Animal Resources at the University of Iowa.

After CMR screening, 12 Reversa mice (7 male, 5 female) with moderate or severe aortic regurgitation (AR^+^), and an additional 12 Reversa mice without aortic regurgitation (AR^-^) underwent comprehensive CMR evaluation of right and left ventricular size and systolic function. Twelve of these mice subsequently entered a study examining cellular and molecular responses to cholesterol-lowering at a later age, which is reported elsewhere[7]. Aortic regurgitation and ventricular function were not examined in that study.

### Screening for aortic regurgitation

**Figure 1:**
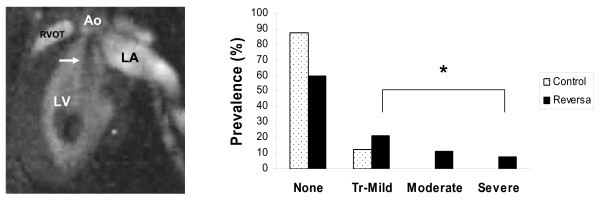
**Magnetic resonance imaging of aortic regurgitation in Reversa mice**. Left panel: Long-axis image of the left ventricle in early diastole before mitral valve opening. Arrow indicates turbulent dephasing of blood caused by diastolic flow across the aortic valve. Video images can be viewed in the on-line video supplement (Additional file 1). Right panel: prevalence of aortic regurgitation, graded by severity, in Reversa mice (*n *= 80) and in all normocholesterolemic control mice (*n *= 40). Tr-Mild = trace or mild. *p = 0.004, Reversa mice *vs*. control mice.

Mice were sedated with midazolam (9 mg/kg) and morphine (4.5 mg/kg) as previously described[9]. Body temperature was maintained with an external heat source. CMR was performed with a Varian Unity/INOVA 4.7 Tesla horizontal bore scanner (Varian, Palo Alto, CA) equipped with a 38 mm quadrature coil (scanning parameters: TR = 6.0 ms; TE = 3.4 ms; flip angle = 15°; 0.2 × 0.2 × 1 mm voxels, 256 × 128 matrix). After collecting localizers, ECG-gated images were acquired at 15–20 frames/cardiac cycle, in coronal oblique planes configured to optimize visualization of the left ventricle along its long-axis and the left ventricular outflow tract (Figure 1 and Additional file 1). This protocol was employed for all 80 Reversa mice and all 40 normocholesterolemic mice. All 120 mice survived the imaging procedure.

The presence and severity of aortic regurgitation were determined by adaptation and modification of a convention introduced by Higgins *et al*[10]. Aortic regurgitation causes dephasing of "white blood" in the left ventricular outflow tract during early diastole (before mitral valve opening). Severity was estimated by the length of the dephasing jet in relation to the valve-apex distance in the long-axis (mild, < 25% of valve-apex distance; moderate, 25–50%; severe: > 50%). Where image quality was sufficient (n = 71 Reversa mice and 32 control mice, respectively), aortic root diameter was measured at the sinotubular junction using electronic calipers.

### Assessment of ventricular structure and function

Hypercholesterolemic Reversa mice with moderate or severe aortic regurgitation (*n *= 12) and an equal number of hypercholesterolic AR^- ^Reversa mice underwent a more thorough evaluation of ventricular size and function. Using the same imaging pulse sequence, images were acquired in 1-mm contiguous planes aligned with the left ventricular short axis. The entire left and right ventricles were imaged in 8–12 slices in 15–20 phases spanning the cardiac cycle.

Left ventricular (LV) and right ventricular (RV) end-diastolic and end-systolic borders were visually identified and electronically planimetered in each slice. End-diastolic and end-systolic volumes, stroke volumes (SV), and left ventricular mass were calculated with software designed for this purpose (Medis^R^, Rotterdam, Netherlands) using Simpson's Rule. Regurgitant fraction (RgF) was calculated using the standard convention: RgF = (LVSV - RVSV)/LVSV

Absence of hemodynamically important aortic valve stenosis was confirmed by visual inspection of aortic valve cusp excursion in the CMR images, and in each case was confirmed by echocardiography[6]. Significant tricuspid and mitral valve regurgitation were excluded by the absence of systolic atrial blood dephasing; pulmonic valve regurgitation was excluded by an absence of retrograde diastolic dephasing arising from the valve region.

### Statistical analysis

Group data are reported as mean ± SE. Analysis of variance was used to compare continuous variables between groups. The proportion of hypercholesterolemic mice with aortic regurgitation was compared to the proportion of normocholesterolemic mice with aortic regurgitation with the Comparison of Two Proportions test[11].

The authors had full access to the data and take responsibility for its integrity. All authors have read and agree to the manuscript.

## Results

**Figure 2:**
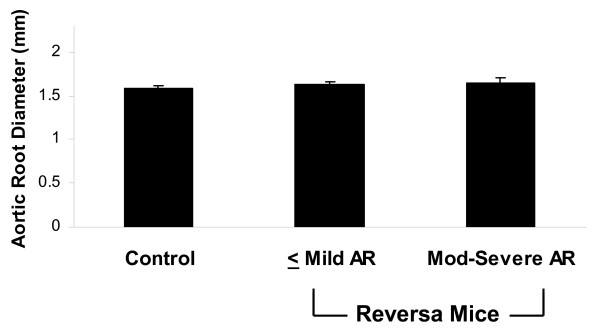
**Aortic root diameter**. Measurements were taken at the level of the sinotubular junction in Control mice (n = 32), Reversa mice without hemodynamically significant aortic regurgitation (< Mild AR, n = 57), and Reversa mice with moderate or severe AR (mod-severe AR, n = 14). p = 0.11 for the comparison.

Screening CMR revealed aortic valve regurgitation in 32 of 80 Reversa mice (18 male, 14 female) fed a high-fat diet. Moderate to severe aortic valve regurgitation occurred in 19% of Reversa mice (Figure 1). Among 40 normocholesterolemic mice, none had moderate or severe aortic valve regurgitation (*p *= 0.008 *vs*. Reversa mice). Trace regurgitation was present in 10% and mild regurgitation was present in 3% of control mice (a combined prevalence of 13%; *p *= 0.004 *vs*. Reversa mice). Aortic root diameter, assessed at the level of the sinotubular junction, was similar between groups of mice (Figure 2).

To assess the impact of chronic volume overload due to aortic regurgitation, 12 Reversa mice with moderate or severe regurgitation (AR^+^) and 12 sex-matched Reversa mice without aortic regurgitation (AR^-^) underwent comprehensive CMR evaluation of the left and right ventricles (Table 1). In AR^+ ^mice, the regurgitant fraction was 0.34 ± 0.04 (vs. 0.02 ± 0.01 in AR^- ^mice, *p *< 0.001). Consequently, left ventricular mass, end-diastolic volume, and stroke volume were higher in AR^+ ^mice, and the left ventricular ejection fraction was impaired.

**Table 1:** Effects of aortic regurgitation on ventricular anatomy and function.

		**AR-**	**AR+**	p
Heart Rate	min^-1^	452 ± 26	445 ± 39	0.88

**Left Ventricle**				
EDV	μL	31.3 ± 1.6	58.4 ± 8.8	0.006
ESV	μL	6.9 ± 1.5	27.4 ± 7.6	0.02
SV	μL	24.4 ± 1.7	31.7 ± 2.9	0.04
EF		0.79 ± 0.04	0.60 ± 0.05	0.007
Mass	mg	48.6 ± 3.3	74.6 ± 7.8	0.005
EDV/Mass		0.68 ± 0.05	0.77 ± 0.05	0.2
Regrg Frac		0.02 ± 0.01	0.34 ± 0.04	< 0.001
**Right Ventricle**				
EDV	μL	31.4 ± 2.2	39.7 ± 3.2	0.046
ESV	μL	7.5 ± 1.1	19.5 ± 1.6	< 0.001
SV	μL	23.9 ± 1.8	20.9 ± 1.9	0.26
EF		0.76 ± 0.03	0.53 ± 0.02	< 0.001

**AR+ **aortic regurgitation present; **AR- **aortic regurgitation absent; **EDV **end-diastolic volume; **SV **stroke volume; **ESV **end-systolic volume; **EF **ejection fraction; **Regrg Frac **regurgitant fraction.

N = 12 in each group.

**Figure 3:**
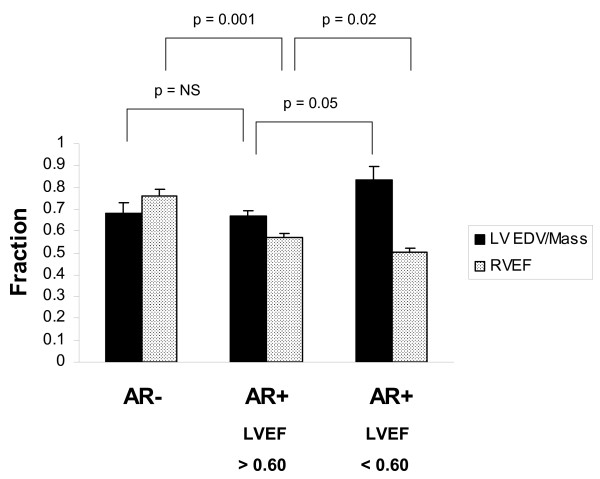
**Effects of left ventricular systolic dysfunction on left ventricular remodeling and right ventricular function**. When LVEF is < 0.60, the end-diastolic volume/mass ratio (LV EDV/Mass) is significantly increased, indicating pathological remodeling, and RVEF is also more significantly impaired.

When left ventricular systolic function was normal or mildly impaired in AR^+ ^mice (defined as ejection fraction ≥ 0.60; *n *= 5), the left ventricular end-diastolic volume/mass ratio was 0.66 ± 0.03, identical to AR^- ^mice. But when impairment of left ventricular systolic function was more severe (defined as ejection fraction < 0.60; n = 7), the left ventricular end-diastolic volume/mass ratio increased to 0.84 ± 0.06, indicating pathological left ventricular dilatation (Figure 3).

AR^+ ^mice manifested right ventricular enlargement and impaired right ventricular systolic function, compared to AR^- ^mice (Table 1). Impairment of right ventricular systolic function was more pronounced in mice with severe left ventricular systolic dysfunction (Figure 3).

## Discussion

The most important findings of this study are that aortic valve regurgitation occurs commonly in hypercholesterolemic *Ldlr*^-/-^/*Apob*^100/100^/*Mttp*^fl/fl^/Mx1Cre^+/+ ^mice, leading to volume overload and pathological remodeling of both ventricles. Whereas hypercholesterolemia can be associated with a more complex "metabolic syndrome", Reversa mice only demonstrate about 10% increase in body mass and do not have elevated blood glucose compared to control mice, at the age reported here[7]. These findings suggest that hypercholesterolemia alone is sufficient to cause aortic regurgitation.

It was previously reported that hypercholesterolemia causes disruption of aortic valve cusp architecture in humans [12] and in Reversa mice [6,7]. This is the likely mechanism for aortic regurgitation since aortic root diameter did not differ significantly between groups of mice in the present study.

In humans, aortic valve regurgitation can also arise from diverse local and systemic processes involving the aorta and/or valve cusps. In retrospective series', conditions such as connective tissue disease, autoimmune or rheumatic disease, infection, trauma, and congenital malformation have all been associated with aortic regurgitation[4]. These other factors may have masked the pathophysiologic importance of hypercholesterolemia, possibly explaining the failure to identify hypercholesterolemia as a risk factor for aortic regurgitation in the Framingham Study[1].

Comprehensive evaluation of a relatively small cohort of patients with familial hypercholesterolemia revealed a very high prevalence of aortic regurgitation: 26% and 80% in heterozygotes and homozygotes, respectively[13]. Similar findings were reported in a younger cohort by Kolansky *et al*[14]. However, since these studies involved patients undergoing cardiac catheterization, selection bias could have been quite significant. The current systematic study shows that hypercholesterolemia does indeed cause aortic regurgitation in mice.

The clinical course of patients with chronic aortic regurgitation is tightly linked to volume overload and the process of cardiac remodeling. Asymptomatic patients with aortic valve regurgitation develop systolic dysfunction at a rate of < 6% per year[15]. Asymptomatic patients with LV dysfunction develop symptoms at a rate of > 25% per year, with an annual mortality > 10%. No medical interventions are known to ameliorate the severity of valve dysfunction, although the hemodynamic impact of aortic valve regurgitation may be mitigated with vasodilators[16,17]. Aortic valve replacement surgery is recommended for patients with impending left ventricular dysfunction or heart failure[15].

Reversa mice offer an attractive model for future studies of the mechanisms by which compensatory cardiac growth in response to volume overload transforms to pathological cardiac remodeling. These mice spontaneously develop the sequelae of chronic left ventricular volume overload observed in humans with aortic regurgitation, and do so in a time frame that permits longitudinal investigation. Because the control group included hypercholesterolemic Reversa mice without aortic regurgitation, there was no doubt that the changes in ventricular structure and function were due to the valvular heart disease.

Right ventricular remodeling and systolic function are gaining acceptance as prognostic indicators in patients with heart disease[18]. Patients with left ventricular systolic dysfunction have a worse prognosis when right ventricular systolic dysfunction is also present[19]. Similarly, patients with mitral valve disease have a worse prognosis after valve surgery if they have concomitant right ventricular systolic dysfunction[20]. Our findings indicate that right ventricular function is impaired in mice with significant aortic regurgitation, and that right ventricular systolic impairment is greater when the left ventricle begins to fail. The prognostic importance of these findings has not been ascertained in the setting of pure aortic regurgitation in mice or humans, and warrants further study.

The findings of this study have methodological implications. Image quality was constrained by the need to image mice with valvular heart disease during deep sedation following a single intraperitoneal injection. However, the data provide a level of "internal validation", by demonstrating that left- and right ventricular stroke volumes are nearly identical in the absence of detectable valve regurgitation, and by reporting relatively narrow parametric ranges in the absence of valvular disease. These attributes combine an experimental model with quantitative noninvasive methods conducive to longitudinal studies designed to investigate disease mechanisms and potential therapies for aortic regurgitation.

The mechanism by which aortic regurgitation spontaneously arises is usually imperfect coaptation of valve cusps during ventricular diastole. Reversa mice may not be unique in this respect. Tanaka *et al *[21] and Aikawa *et al *[22] reported anecdotal evidence of aortic regurgitation in *Apoe*^-/- ^mice. Jordan *et al *reported anecdotal evidence of aortic valve regurgitation in mice with mucopolysaccharidosis[23]. The current study is distinct from these earlier studies in that it assesses the causal role of hypercholesterolemia in aortic valve regurgitation in a systematic fashion, and provides quantitative determination of the severity of valve dysfunction and its impact on ventricular structure and function.

This study has several limitations. Reversa mice had very high cholesterol levels, which are seen clinically only in patients with homozygous mutations in the low-density lipoprotein receptor. The results are reported for a single time point – age 7.5 months. The long term risk of overt heart failure or death from aortic regurgitation in this mouse model has not yet been ascertained. A previous study reported that Reversa mice develop hemodynamically significant aortic valve stenosis by age 18 – 22 months.[6] In that study, mice did not undergo CMR to assess the presence or severity of aortic regurgitation. It is not yet known whether individual mice undergo evolution of predominant valvular functional phenotype as they age. Also, we have not yet addressed whether the progression of aortic valve regurgitation can be retarded by reversal of the hypercholesterolemia. Future studies are warranted to address these questions.

## Conclusion

Hypercholesterolemia causes aortic valve regurgitation with moderate prevalence in mice. When present, aortic valve regurgitation causes volume overload and pathological remodeling of both ventricles.

## Competing interests

The authors declare that they have no competing interests.

## Authors' contributions

CJB acquired and analyzed CMR data. JDM bred and characterized hypercholesterolemic mice and contributed importantly to experimental design. KLM acquired and analyzed CMR data. DRT designed the pulse sequences for assessment of valve function. SGY created the original strain of hypercholesterolemic mice, provided founders, and critically edited the manuscript. DDH participated importantly in the experimental design and critically edited the manuscript. RMW designed the study, oversaw data acquisition and analysis, and participated importantly in the writing of the manuscript.

## Supplementary Material

Additional file 1 **Video Supplement**. Video file containing cine CMR images from a Reversa mouse with severe aortic regurgitation. Click here for file
